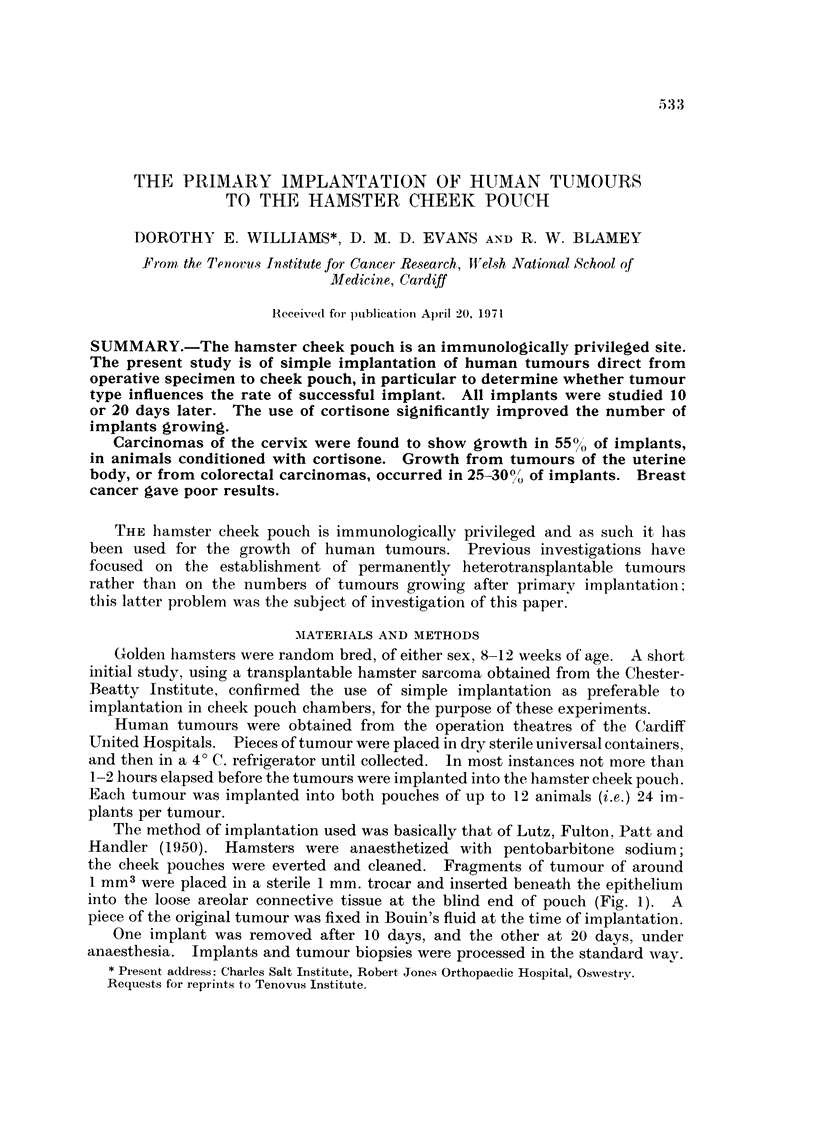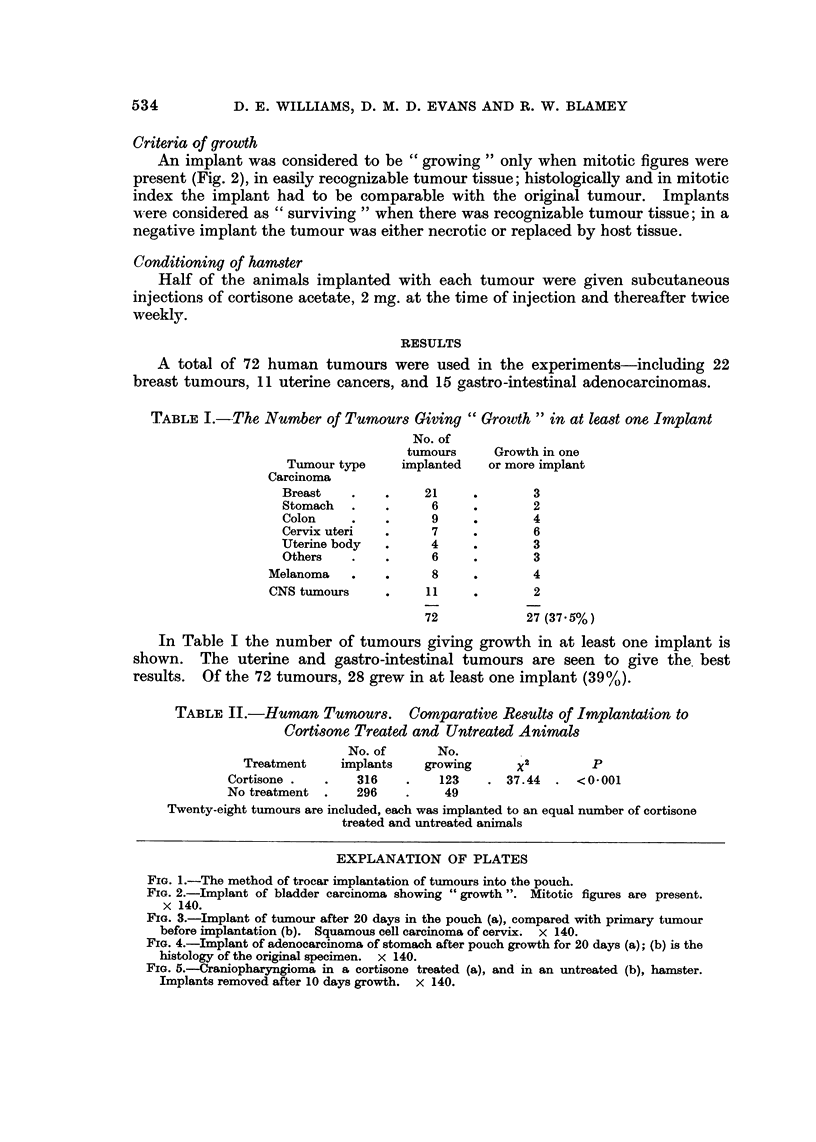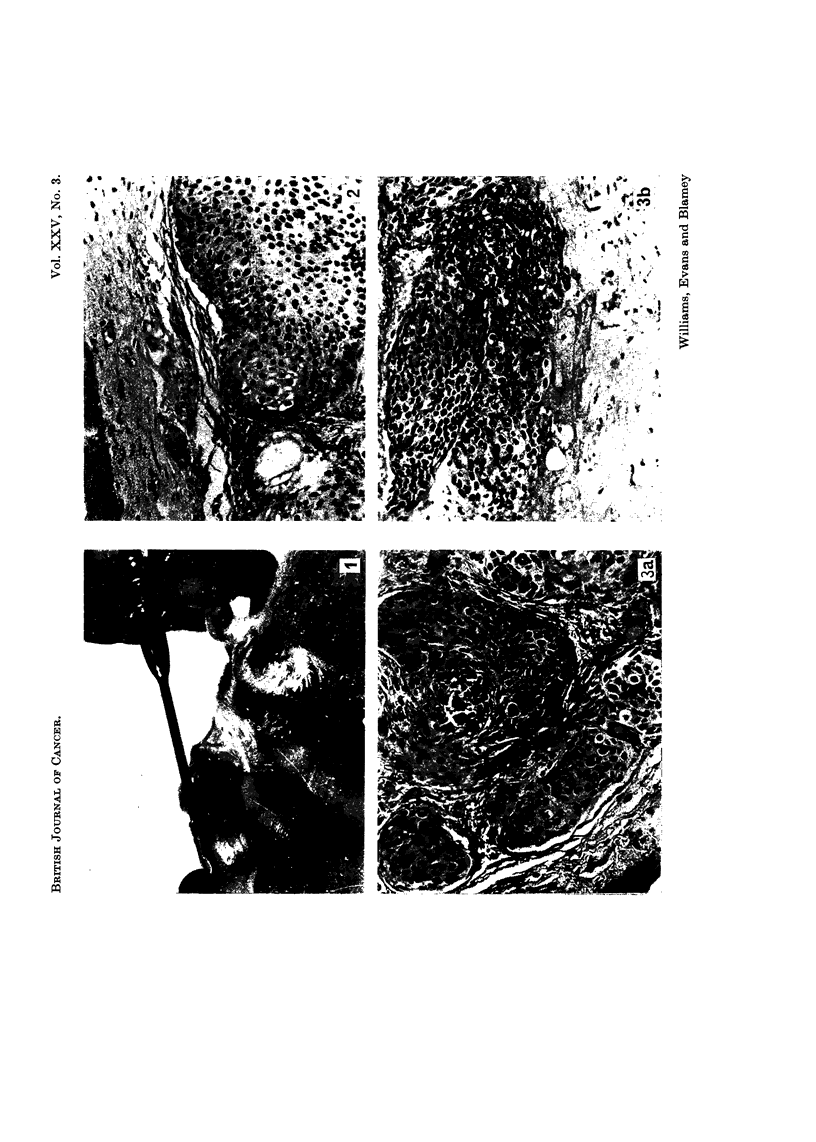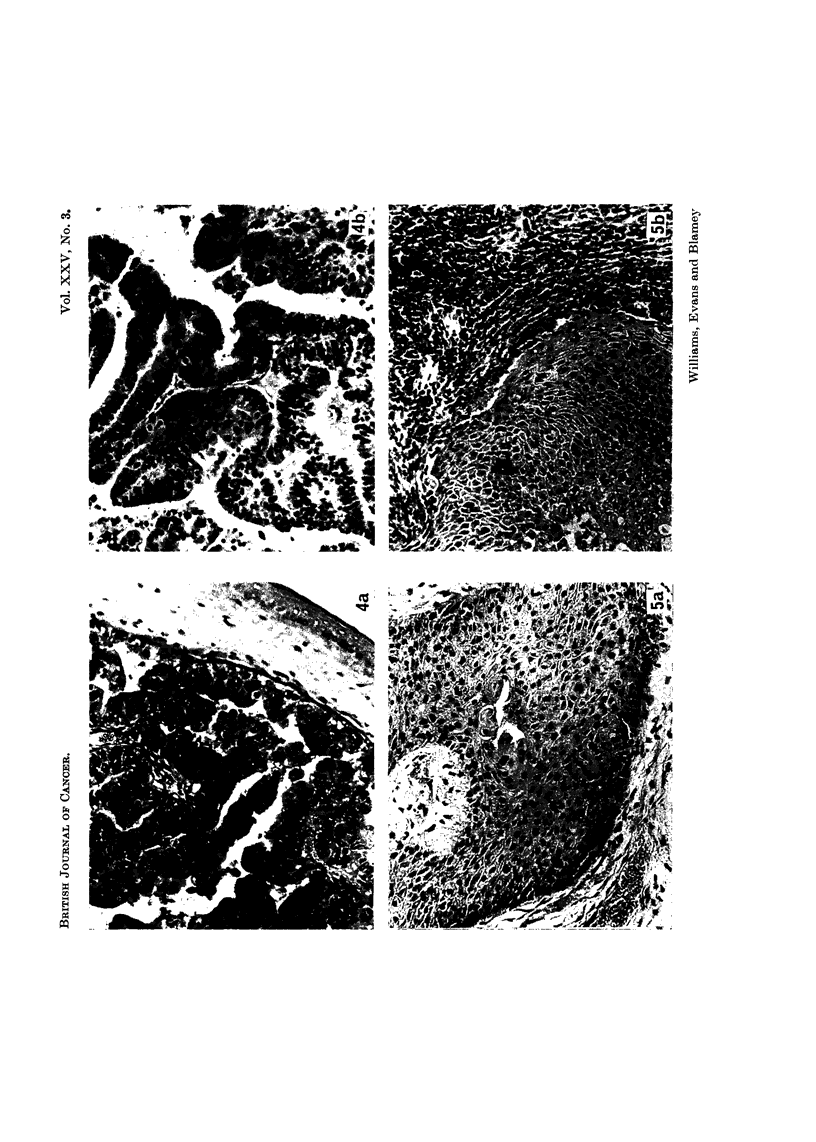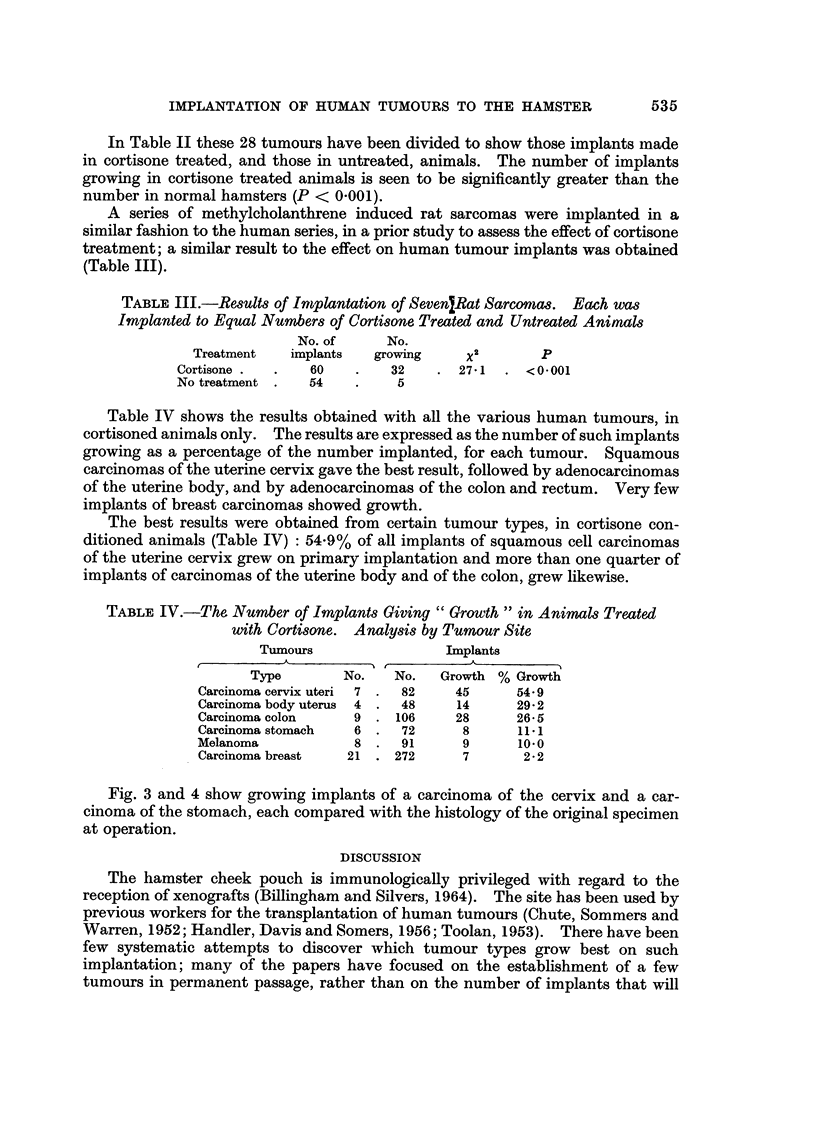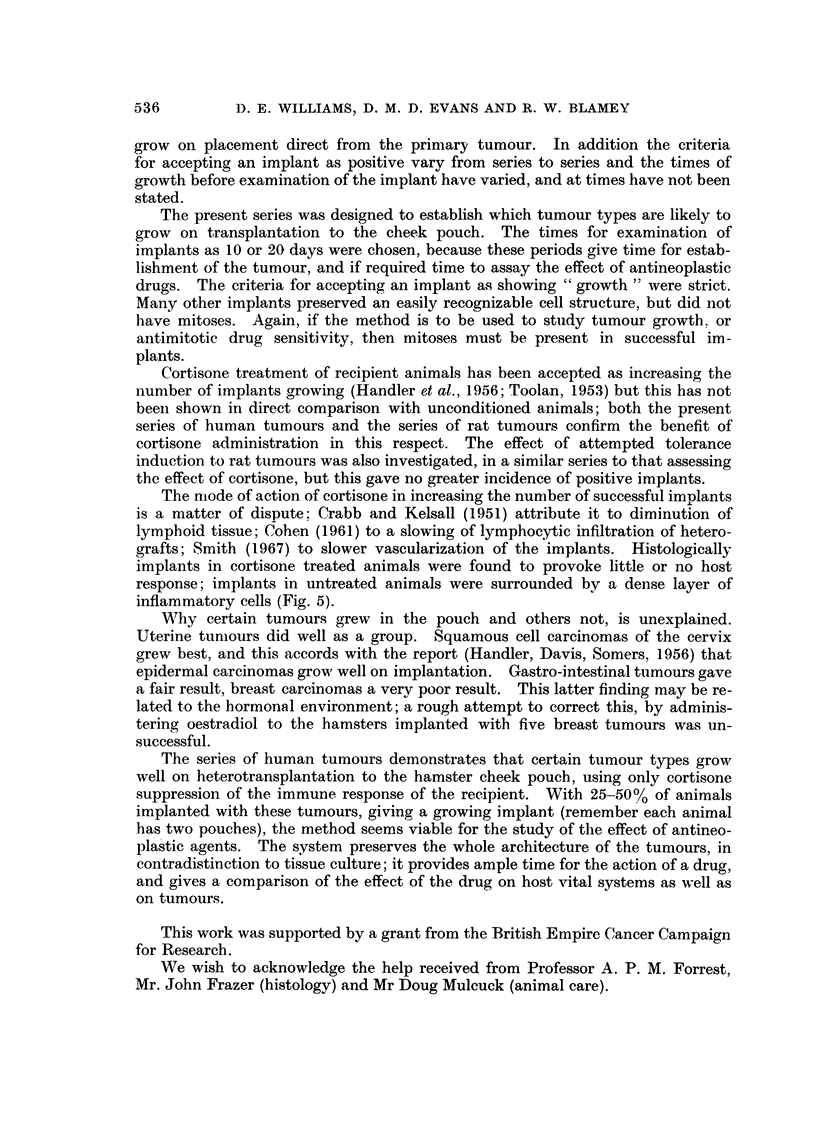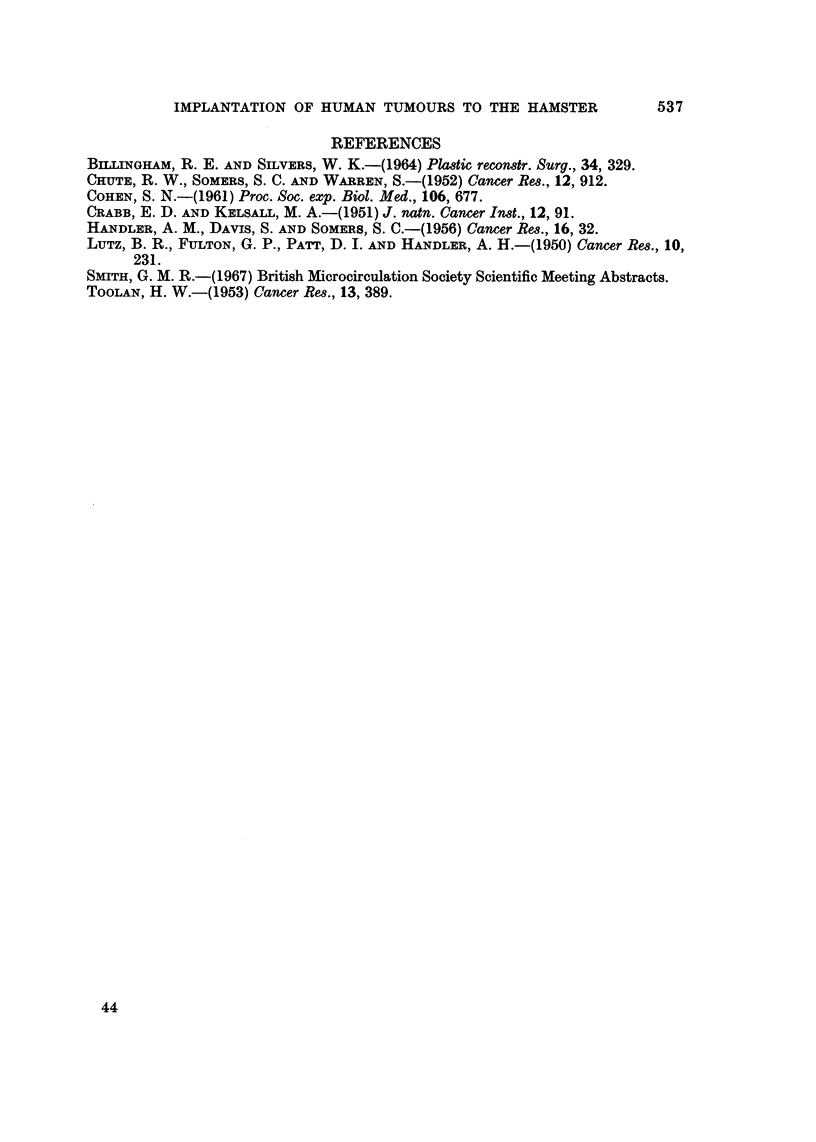# The Primary Implantation of Human Tumours to the Hamster Cheek Pouch

**DOI:** 10.1038/bjc.1971.68

**Published:** 1971-09

**Authors:** Dorothy E. Williams, D. M. D. Evans, R. W. Blamey

## Abstract

**Images:**


### THE PRIMARY IMPLANTATION OF HUMAN TUMOURS TO THE HAMSTER CHEEK POUCH

DOROTHY E. WILLIAMS*, D. M. D. EVANS AND R. W. BLAMEY

*From the Tenovus Institute for Cancer Research, Welsh National School of Medicine, Cardiff*

Received for publication April 20, 1971

**SUMMARY.—The hamster cheek pouch is an immunologically privileged site. The present study is of simple implantation of human tumours direct from operative specimen to cheek pouch, in particular to determine whether tumour type influences the rate of successful implant. All implants were studied 10 or 20 days later. The use of cortisone significantly improved the number of implants growing.**

**Carcinomas of the cervix were found to show growth in 55% of implants, in animals conditioned with cortisone. Growth from tumours of the uterine body, or from colorectal carcinomas, occurred in 25–30% of implants. Breast cancer gave poor results.**

THE hamster cheek pouch is immunologically privileged and as such it has been used for the growth of human tumours. Previous investigations have focused on the establishment of permanently heterotransplantable tumours rather than on the numbers of tumours growing after primary implantation; this latter problem was the subject of investigation of this paper.

### MATERIALS AND METHODS

Golden hamsters were random bred, of either sex, 8–12 weeks of age. A short initial study, using a transplantable hamster sarcoma obtained from the Chester-Beatty Institute, confirmed the use of simple implantation as preferable to implantation in cheek pouch chambers, for the purpose of these experiments.

Human tumours were obtained from the operation theatres of the Cardiff United Hospitals. Pieces of tumour were placed in dry sterile universal containers, and then in a 4° C. refrigerator until collected. In most instances not more than 1–2 hours elapsed before the tumours were implanted into the hamster cheek pouch. Each tumour was implanted into both pouches of up to 12 animals (*i.e.*) 24 implants per tumour.

The method of implantation used was basically that of Lutz, Fulton, Patt and Handler (1950). Hamsters were anaesthetized with pentobarbitone sodium; the cheek pouches were everted and cleaned. Fragments of tumour of around 1 mm$^3$ were placed in a sterile 1 mm. trocar and inserted beneath the epithelium into the loose areolar connective tissue at the blind end of pouch (Fig. 1). A piece of the original tumour was fixed in Bouin's fluid at the time of implantation.

One implant was removed after 10 days, and the other at 20 days, under anaesthesia. Implants and tumour biopsies were processed in the standard way.

* Present address: Charles Salt Institute, Robert Jones Orthopaedic Hospital, Oswestry.

Requests for reprints to Tenovus Institute.

*Criteria of growth*

An implant was considered to be " growing " only when mitotic figures were present (Fig. 2), in easily recognizable tumour tissue; histologically and in mitotic index the implant had to be comparable with the original tumour. Implants were considered as " surviving " when there was recognizable tumour tissue; in a negative implant the tumour was either necrotic or replaced by host tissue.

*Conditioning of hamster*

Half of the animals implanted with each tumour were given subcutaneous injections of cortisone acetate, 2 mg. at the time of injection and thereafter twice weekly.

### RESULTS

A total of 72 human tumours were used in the experiments—including 22 breast tumours, 11 uterine cancers, and 15 gastro-intestinal adenocarcinomas.

TABLE I.—*The Number of Tumours Giving " Growth " in at least one Implant*

| Tumour type | No. of tumours implanted | Growth in one or more implant |
|---|---|---|
| Carcinoma | | |
| Breast | 21 | 3 |
| Stomach | 6 | 2 |
| Colon | 9 | 4 |
| Cervix uteri | 7 | 6 |
| Uterine body | 4 | 3 |
| Others | 6 | 3 |
| Melanoma | 8 | 4 |
| CNS tumours | 11 | 2 |
| | 72 | 27 (37·5%) |

In Table I the number of tumours giving growth in at least one implant is shown. The uterine and gastro-intestinal tumours are seen to give the best results. Of the 72 tumours, 28 grew in at least one implant (39%).

TABLE II.—*Human Tumours. Comparative Results of Implantation to Cortisone Treated and Untreated Animals*

| Treatment | No. of implants | No. growing | $\chi^2$ | $P$ |
|---|---|---|---|---|
| Cortisone | 316 | 123 | 37.44 | <0·001 |
| No treatment | 296 | 49 | | |

Twenty-eight tumours are included, each was implanted to an equal number of cortisone treated and untreated animals

### EXPLANATION OF PLATES

FIG. 1.—The method of trocar implantation of tumours into the pouch.

FIG. 2.—Implant of bladder carcinoma showing " growth ". Mitotic figures are present. × 140.

FIG. 3.—Implant of tumour after 20 days in the pouch (a), compared with primary tumour before implantation (b). Squamous cell carcinoma of cervix. × 140.

FIG. 4.—Implant of adenocarcinoma of stomach after pouch growth for 20 days (a); (b) is the histology of the original specimen. × 140.

FIG. 5.—Craniopharyngioma in a cortisone treated (a), and in an untreated (b), hamster. Implants removed after 10 days growth. × 140.

Williams, Evans and Blamey

Williams, Evans and Blamey

In Table II these 28 tumours have been divided to show those implants made in cortisone treated, and those in untreated, animals. The number of implants growing in cortisone treated animals is seen to be significantly greater than the number in normal hamsters ($P < 0{\cdot}001$).

A series of methylcholanthrene induced rat sarcomas were implanted in a similar fashion to the human series, in a prior study to assess the effect of cortisone treatment; a similar result to the effect on human tumour implants was obtained (Table III).

TABLE III.—*Results of Implantation of Seven Rat Sarcomas. Each was Implanted to Equal Numbers of Cortisone Treated and Untreated Animals*

| Treatment | No. of implants | No. growing | $\chi^2$ | $P$ |
|---|---|---|---|---|
| Cortisone | 60 | 32 | 27·1 | <0·001 |
| No treatment | 54 | 5 | | |

Table IV shows the results obtained with all the various human tumours, in cortisoned animals only. The results are expressed as the number of such implants growing as a percentage of the number implanted, for each tumour. Squamous carcinomas of the uterine cervix gave the best result, followed by adenocarcinomas of the uterine body, and by adenocarcinomas of the colon and rectum. Very few implants of breast carcinomas showed growth.

The best results were obtained from certain tumour types, in cortisone conditioned animals (Table IV) : 54·9% of all implants of squamous cell carcinomas of the uterine cervix grew on primary implantation and more than one quarter of implants of carcinomas of the uterine body and of the colon, grew likewise.

TABLE IV.—*The Number of Implants Giving " Growth " in Animals Treated with Cortisone. Analysis by Tumour Site*

| Tumours | | Implants | | |
|---|---|---|---|---|
| Type | No. | No. | Growth | % Growth |
| Carcinoma cervix uteri | 7 | 82 | 45 | 54·9 |
| Carcinoma body uterus | 4 | 48 | 14 | 29·2 |
| Carcinoma colon | 9 | 106 | 28 | 26·5 |
| Carcinoma stomach | 6 | 72 | 8 | 11·1 |
| Melanoma | 8 | 91 | 9 | 10·0 |
| Carcinoma breast | 21 | 272 | 7 | 2·2 |

Fig. 3 and 4 show growing implants of a carcinoma of the cervix and a carcinoma of the stomach, each compared with the histology of the original specimen at operation.

### DISCUSSION

The hamster cheek pouch is immunologically privileged with regard to the reception of xenografts (Billingham and Silvers, 1964). The site has been used by previous workers for the transplantation of human tumours (Chute, Sommers and Warren, 1952; Handler, Davis and Somers, 1956; Toolan, 1953). There have been few systematic attempts to discover which tumour types grow best on such implantation; many of the papers have focused on the establishment of a few tumours in permanent passage, rather than on the number of implants that will grow on placement direct from the primary tumour. In addition the criteria for accepting an implant as positive vary from series to series and the times of growth before examination of the implant have varied, and at times have not been stated.

The present series was designed to establish which tumour types are likely to grow on transplantation to the cheek pouch. The times for examination of implants as 10 or 20 days were chosen, because these periods give time for establishment of the tumour, and if required time to assay the effect of antineoplastic drugs. The criteria for accepting an implant as showing " growth " were strict. Many other implants preserved an easily recognizable cell structure, but did not have mitoses. Again, if the method is to be used to study tumour growth, or antimitotic drug sensitivity, then mitoses must be present in successful implants.

Cortisone treatment of recipient animals has been accepted as increasing the number of implants growing (Handler *et al.*, 1956; Toolan, 1953) but this has not been shown in direct comparison with unconditioned animals; both the present series of human tumours and the series of rat tumours confirm the benefit of cortisone administration in this respect. The effect of attempted tolerance induction to rat tumours was also investigated, in a similar series to that assessing the effect of cortisone, but this gave no greater incidence of positive implants.

The mode of action of cortisone in increasing the number of successful implants is a matter of dispute; Crabb and Kelsall (1951) attribute it to diminution of lymphoid tissue; Cohen (1961) to a slowing of lymphocytic infiltration of heterografts; Smith (1967) to slower vascularization of the implants. Histologically implants in cortisone treated animals were found to provoke little or no host response; implants in untreated animals were surrounded by a dense layer of inflammatory cells (Fig. 5).

Why certain tumours grew in the pouch and others not, is unexplained. Uterine tumours did well as a group. Squamous cell carcinomas of the cervix grew best, and this accords with the report (Handler, Davis, Somers, 1956) that epidermal carcinomas grow well on implantation. Gastro-intestinal tumours gave a fair result, breast carcinomas a very poor result. This latter finding may be related to the hormonal environment; a rough attempt to correct this, by administering oestradiol to the hamsters implanted with five breast tumours was unsuccessful.

The series of human tumours demonstrates that certain tumour types grow well on heterotransplantation to the hamster cheek pouch, using only cortisone suppression of the immune response of the recipient. With 25–50% of animals implanted with these tumours, giving a growing implant (remember each animal has two pouches), the method seems viable for the study of the effect of antineoplastic agents. The system preserves the whole architecture of the tumours, in contradistinction to tissue culture; it provides ample time for the action of a drug, and gives a comparison of the effect of the drug on host vital systems as well as on tumours.

This work was supported by a grant from the British Empire Cancer Campaign for Research.

We wish to acknowledge the help received from Professor A. P. M. Forrest, Mr. John Frazer (histology) and Mr Doug Mulcuck (animal care).

### REFERENCES

BILLINGHAM, R. E. AND SILVERS, W. K.—(1964) *Plastic reconstr. Surg.*, **34**, 329.

CHUTE, R. W., SOMERS, S. C. AND WARREN, S.—(1952) *Cancer Res.*, **12**, 912.

COHEN, S. N.—(1961) *Proc. Soc. exp. Biol. Med.*, **106**, 677.

CRABB, E. D. AND KELSALL, M. A.—(1951) *J. natn. Cancer Inst.*, **12**, 91.

HANDLER, A. M., DAVIS, S. AND SOMERS, S. C.—(1956) *Cancer Res.*, **16**, 32.

LUTZ, B. R., FULTON, G. P., PATT, D. I. AND HANDLER, A. H.—(1950) *Cancer Res.*, **10**, 231.

SMITH, G. M. R.—(1967) British Microcirculation Society Scientific Meeting Abstracts.

TOOLAN, H. W.—(1953) *Cancer Res.*, **13**, 389.